# Targeted therapy against recurrent anti-synthetase syndrome associated interstitial lung disease after bilateral lung transplantation guided by transcriptomic analyses: a case-based study

**DOI:** 10.3389/fimmu.2026.1857240

**Published:** 2026-07-22

**Authors:** Shiyao Wang, Qingya Shi, Wenxiu Xu, Haibo Li, Lili Zhu, Mengyin Chen, Shifeng Li, Lijuan Guo, Li Zhao, Bin Xing, Zhibo Liu, Yi Zhang, Shiwei Qumu, Yinan Hu, Lingyan You, Quanguo Li, Jing Sun, Sizhao Li, Min Liu, Ling Zhao, Ye Cui, Huaping Dai, Wenhui Chen

**Affiliations:** 1 National Center for Respiratory Medicine; State Key Laboratory of Respiratory Health and Multimorbidity; National Clinical Research Center for Respiratory Diseases; Institute of Respiratory Medicine, Chinese Academy of Medical Sciences & Peking Union Medical College; Department of Pulmonary and Critical Care Medicine, Center of Respiratory Medicine, China-Japan Friendship Hospital, Beijing, China; 2 National Center for Respiratory Medicine; State Key Laboratory of Respiratory Health and Multimorbidity; National Clinical Research Center for Respiratory Diseases; Institute of Respiratory Medicine, Chinese Academy of Medical Sciences & Peking Union Medical College; Department of Lung Transplantation, Center of Respiratory Medicine, China-Japan Friendship Hospital, Beijing, China; 3 School of Medicine, Tsinghua Medicine, Tsinghua University, Beijing, China; 4 Center of Respiratory and Pulmonary Vascular Disease, Fuwai Hospital, National Center for Cardiovascular Disease, Chinese Academy of Medical Sciences and Peking Union Medical College, Beijing, China; 5 Department of Pulmonary and Critical Care Medicine, Weifang Respiratory Disease Hospital, Weifang No. 2 Renmin Hospital, Weifang, Shandong, China; 6 Department of Rheumatology, China-Japan Friendship Hospital, Beijing, China; 7 Department of Radiology, China-Japan Friendship Hospital, Beijing, China; 8 Department of Pathology, China-Japan Friendship Hospital, Beijing, China; 9 Department of Immunology, School of Basic Medical Sciences, Capital Medical University, Beijing, China

**Keywords:** anti-synthetase syndrome, interferon and JAK-STAT pathways, interstitial lung disease, post lung transplantation, transcriptomic analysis

## Abstract

**Background:**

Anti-synthetase syndrome (ASS) associated interstitial lung disease (ILD) usually responds to immunosuppressive therapy, but recurrence is common. We report a 58-year-old man with ASS-ILD who developed recurrent ILD within one year after bilateral lung transplantation (LTx). Despite triple immunosuppression (glucocorticoids, tacrolimus, mycophenolate mofetil), systemic inflammation persisted. This case represents an *in vivo* model of ASS-ILD recurrence, warranting further investigation into underlying mechanisms and novel therapeutic strategies.

**Methods:**

Peripheral blood mononuclear cells (PBMCs) were collected at 56 and 84 weeks post-transplant for single-cell RNA sequencing (scRNA-seq), while lung tissue was analyzed via spatial transcriptomics. Control data came from five naïve ASS-ILD patients and two clinically stable connective tissue disease associated ILD (CTD-ILD) patients post-LTx. Differential gene expression and pathway enrichment analyses were performed to identify therapeutic targets.

**Results:**

Exploratory PBMC scRNA-seq analysis suggested enrichment of interferon-, interleukin- and JAK-STAT-related signaling programs in circulating monocytes and neutrophils. Based on this immune activation profile, a multidrug treatment adjustment was implemented, including short-term glucocorticoid augmentation, replacement of mycophenolate mofetil with Janus kinase inhibitor tofacitinib, and replacement of tacrolimus with cyclosporine A. Following treatment adjustment, systemic inflammatory markers declined and interstitial lesions in both lungs were markedly alleviated on imaging. Subsequent spatial transcriptomic analysis of lung tissue revealed persistent interferon-related signaling and identified pro-fibrotic transcriptional programs in alveolar macrophages and transitional type II alveolar cells. Functional enrichment suggested a potential association between systemic inflammatory activation and localized fibrotic remodeling within the lung microenvironment.

**Conclusions:**

This case illustrates the potential utility of scRNA-seq and spatial transcriptomics for characterizing immune-related transcriptional programs in recurrent ASS-ILD after LTx. Transcriptomic profiling informed therapeutic decision-making in this complex clinical setting, and clinical improvement was observed following treatment adjustment. These findings generate exploratory insights into potential therapeutic targets in recurrent ASS-ILD.

## Background

Anti-synthetase syndrome (ASS) is a specific subtype of idiopathic inflammatory myopathy (IIM) characterized by the presence of autoantibodies targeting transfer RNA (tRNA) synthetases. These include, but are not limited to, anti-histidyl (Jo-1), anti-threonyl (PL-7), anti-alanyl (PL-12), anti-isoleucyl (OJ), and anti-glycyl (EJ) tRNA synthetase antibodies (1). The main clinical manifestations of ASS include interstitial lung disease (ILD), myositis, rashes such as mechanic’s hand and Gottron’s papules, and arthritis, with ILD being the main factor affecting the patient’s prognosis (2). Most ASS respond well to glucocorticoids and immunosuppressive agents, with cyclophosphamide, calcineurin inhibitors (CNIs), and mycophenolate being the commonly used immunosuppressive treatments.

However, despite aggressive immunosuppressive therapy in some patients, the ILD may continue to progress, showing pattern of progressive pulmonary fibrosis and eventually resulting in respiratory failure. Lung transplantation (LTx) may be a therapeutic option for these patients with end-stage ASS associated ILD (ASS-ILD) (3–5). Due to the need for continuous use of immunosuppressive drugs after LTx, the condition of ASS may also achieve stable control. Here, we report a case of a patient who underwent bilateral LTx due to ASS-ILD. The patient’s ILD recurred within one year after LTx, but the condition was brought back under control by selecting specific targeted drugs through the investigation of systemic inflammatory features.

## Case report

A 58-year-old Chinese male with no smoking history began experiencing cough and exertional dyspnea six years before bilateral LTx. A chest computed tomography (CT) scan at a local hospital indicated ILD, but he received no treatment. Four years later, his symptoms worsened, and he developed mechanic’s hands and Gottron’s papules. Testing revealed strong positivity for anti-PL-7 antibodies, confirming a diagnosis of ASS-ILD. Despite treatment with prednisone, cyclophosphamide, and pirfenidone, the patient’s lung function continued to deteriorate (from 2 years to 1 year before LTx). The immunosuppressive regimen was subsequently adjusted to prednisone, tacrolimus, and mycophenolate mofetil (MMF) (from 1 year to 1 month before LTx). However, the decline in lung function remained uncontrolled, with chest imaging showing fibrotic nonspecific interstitial pneumonia (NSIP) pattern. After careful evaluation, the patient underwent bilateral LTx on November 9, 2020, with veno-venous extracorporeal membrane oxygenation support.

Post-transplant, the patient received triple immunosuppressive therapy with glucocorticoids, tacrolimus (target trough concentration: 9–11 ng/ml), and mycophenolic acid (MPA) (MMF 1g/d). His lung function and exercise tolerance gradually improved. In the early post-transplant period, he had infections with filamentous fungi, *Enterococcus faecium*, and methicillin-sensitive *Staphylococcus aureus*, but recovered after appropriate treatment. However, by the 38th week post-transplant, he developed persistent fever, and a chest CT showed multiple ground-glass opacities and consolidation, indicating interstitial infiltration. Laboratory tests revealed elevated white blood cell (WBC) count (13.68×10^9^/L, normal range 3.50-9.50 ×10^9^/L), serum creatine kinase (CK) (2729 U/L, normal range 26–200 U/L), C-reactive protein (CRP) (8.98mg/L, normal range <0.8mg/L), and ferritin (976 ng/ml, normal range 11.0-306.8 ng/ml). Repeat myositis-specific autoantibody (MSA) testing showed strong positivity for anti-PL-7 antibodies. Panel reactive antibodies (PRA) testing was negative. Pathogen testing of bronchioalveolar lavage fluid (BALF) was negative. TBLB performed at 39^th^ week showed no evidence of acute cellular rejection (A0B0 according to ISHLT criteria), malignancy, or granulomatous inflammation, thereby arguing against acute graft rejection. Taken together, a diagnosis of ASS recurrence with ILD and myositis was established.

Then, the patient was treated with high-dose glucocorticoids (methylprednisolone (MP) 160 mg/day for 3 days, followed by tapering), which normalized his temperature. However, after tapering the glucocorticoids, his temperature rose again, and laboratory tests showed persistently elevated WBC count (39.87×10^9^/L at 46 weeks), CK (2916 U/L at 44 weeks), CRP (50.22 mg/L at 47 weeks), and ferritin (1743.9 ng/ml at 54 weeks). Chest CT scans revealed worsening interstitial lung lesions, and the PaO_2_/FiO_2_ ratio declined to 248 mmHg at 63 weeks. Additionally, the patient experienced several adverse effects from glucocorticoid treatment, including upper gastrointestinal bleeding, pulmonary infections with *Aspergillus fumigatus*, *Pseudomonas aeruginosa*, and *Corynebacterium striatum*, and urinary tract infections caused by *Klebsiella* species.

The choice of anti-inflammatory treatment was challenging. High-dose glucocorticoids were effective but unsuitable for long-term use, and tacrolimus and MPA failed to control the patient’s systemic inflammation. Identifying the patient’s specific inflammatory characteristics was crucial for selecting the right treatment. Based on previous research (6), we performed single-cell RNA sequencing (scRNA-seq) of peripheral blood mononuclear cells (PBMCs) to guide therapy adjustments. After modifying the treatment based on initial scRNA-seq results, we conducted a follow-up PBMC scRNA-seq analysis to assess changes in immune characteristics. Additionally, spatial transcriptomic analysis was performed on lung biopsy tissue from the patient’s ASS-ILD recurrence to explore how peripheral blood inflammatory states were reflected in lung tissue.

**Figure 1:**
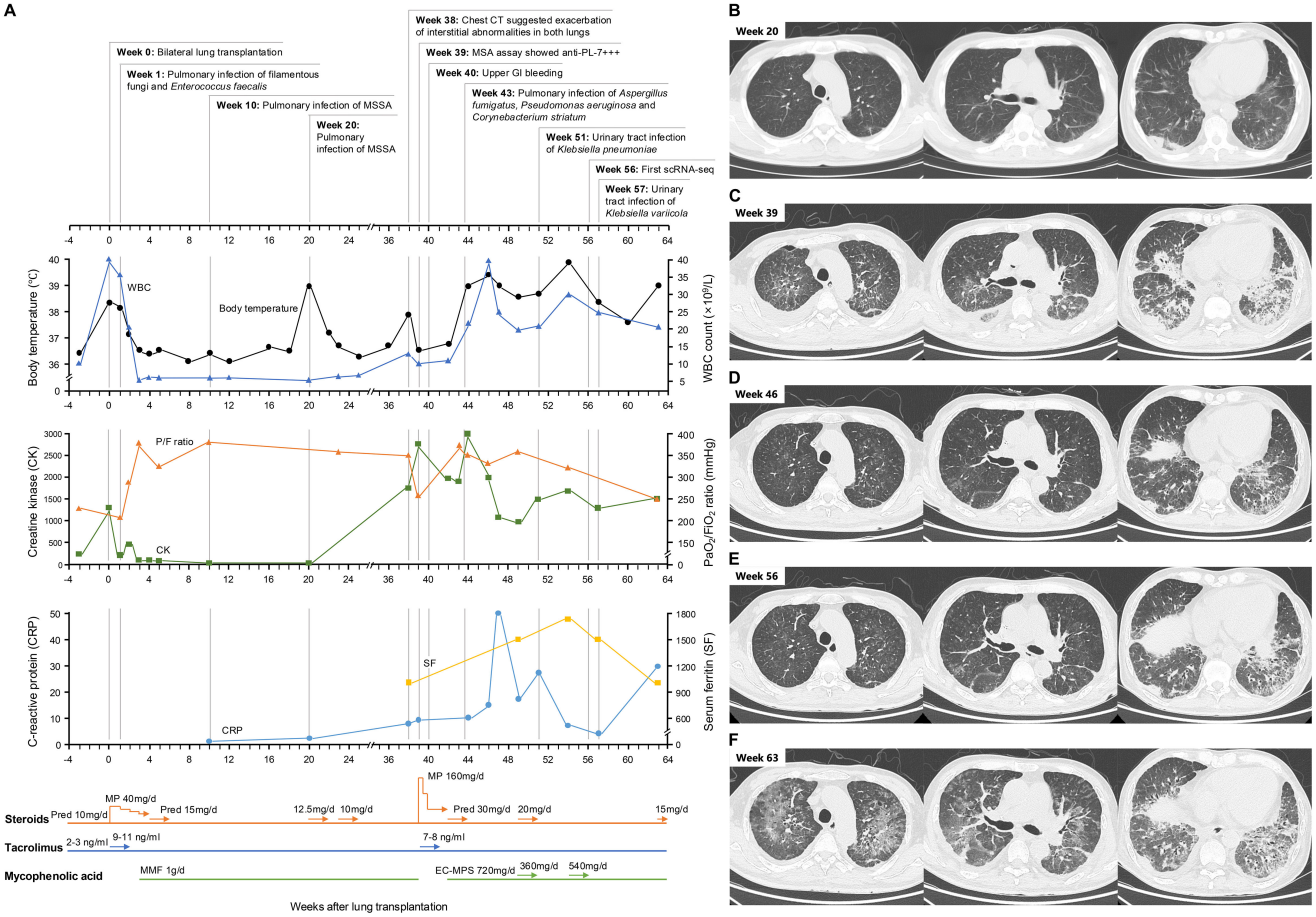
Clinical manifestations and immunosuppressive regimen within 64 weeks after LTx. **(A)** Key clinical events, changes in laboratory parameters, and treatment regimens. Accompanying chest CT imaging changes at 20 **(B)**, 39 **(C)**, 46 **(D)**, 56 **(E)**, and 63 **(F)** weeks post-transplantation. MSSA, methicillin-susceptible *Staphylococcus aureus*; MSA, myositis specific autoantibody; GI, gastrointestinal; scRNA-seq, single-cell RNA sequencing; WBC, white blood cell; Pred, prednisone; MP, methylprednisolone; MMF, mycophenolate mofetil; EC-MPS, enteric-coated mycophenolate sodium.

The clinical manifestations, key events, chest imaging changes, and immunosuppressive regimen within 64 weeks post-transplant are presented in **Figure 1**.

## Methods

### Sample collection and control data sources

Peripheral venous blood (10 mL) was collected at 56 and 84 weeks post-transplant (before and after treatment adjustment). PBMCs were isolated immediately using Ficoll-Hypaque density-gradient centrifugation, with cell viability exceeding 90% for all samples. For spatial transcriptomic analysis, specimens were obtained from TBLB performed at 39 weeks post-transplant. The timing of sample collection relative to major clinical events, and treatment modifications of the patient is summarized in **Supplementary Figure S2**. The control PBMC scRNA-seq datasets were derived from five naïve ASS-ILD patients who did not undergo treatment and LTx (previously published (6); GSE190510) and two clinically stable connective tissue disease associated ILD (CTD-ILD) patients post-LTx (samples collected using the same protocol as described above; GSE324889). Control pathological specimens for spatial transcriptomic analysis were derived from a transbronchial lung cryobiopsy (TBLC) specimen obtained from one of the five naïve ASS-ILD patients (Patient 4 in **Supplementary Table S1**). Detailed demographic, serological, radiological, pulmonary function, pathological, and treatment information for all control patients are provided in **Supplementary Table S1**, **S2** and **Supplementary Figure S3**, **S4**. This study was approved by the Ethics Committee of the China-Japan Friendship Hospital, China (2019-123-K85), and written informed consent was obtained from all participants.

### scRNA-seq of PBMCs

Single-cell suspensions (300–600 viable cells/μL, Count Star) were processed using the Chromium Next GEM Single Cell 5′ Library and Gel Bead Kit v1.1 (10X Genomics, 1000165) and Chromium Next GEM Chip G Single Cell Kit (10X Genomics, 1000127). Approximately 10,000 cells per channel were loaded onto a Chromium Controller to generate single-cell gel beads-in-emulsion (GEMs). Cells were suspended in phosphate-buffered saline (PBS) with 0.04% bovine serum albumin (BSA), yielding ~5,000 recovered cells per channel. Cell lysis and RNA barcoding were performed through reverse transcription (RT) within GEMs using a S1000TM Touch Thermal Cycler (Bio Rad): 53 °C for 45 minutes, 85 °C for 5 minutes, and 4 °C hold. Complementary DNA (cDNA) was amplified and quality-checked using an Agilent 4200 (CapitalBio Technology, Beijing). scRNA-seq libraries were constructed following the manufacturer’s protocol and sequenced on an Illumina Novaseq6000 with a paired-end 150bp (PE150) strategy, achieving >100,000 reads per cell (CapitalBio Technology, Beijing).

### Quality control, doublet removal, and batch effect correction for scRNA-seq data

In this study, scRNA-seq analyses were performed in two distinct groups using Seurat v5 (7). For the first group of five naïve ASS-ILD patients, two clinically stable CTD-ILD patients post-LTx and a pre-treatment adjustment patient, cell retention criteria were defined as follows: (1) cells with detected gene expression between the 2.5th and 97.5th percentiles; (2) cells with total UMIs between the 5th and 95th percentiles; (3) cells with mitochondrial gene content under 10% of their total gene profile. Doublet removal was performed using DoubletFinder (v2.0.4), and batch effect correction was applied with “Harmony” method. For the second group (before vs. after treatment adjustment), cell retention filtering, doublet removal and batch effect correction were performed using the same procedures as described for the first group.

### Cell clustering and annotation

High-quality cells were subjected to clustering using the FindNeighbors and FindClusters functions. Optimal number of principal components (PCs) were selected via elbow plot. Subsequent dimension reduction was achieved through Uniform Manifold Approximation and Projection (UMAP) by employing the R package RunUMAP. The visualization of the cell clustering was performed using the DimPlot function. Cells were then annotated manually based on the expression of established canonical markers.

### Differential gene expression analysis and pathway enrichment

Differentially expressed genes (DEGs) were identified using the FindMarkers function built in Seurat v5, employing the MAST test with a minimum detection rate (min.pct) threshold set at 0.2. Only the genes with the adjusted *p-value* less than 0.05 and average log_2_FoldChange higher than 1 were considered as the most significant DEGs, which were further selected for the enrichment analysis of Reactome pathways via ReactomePA (8) (version 1.38.0). Given the single-patient design and limited size of the control groups, all differential expression and enrichment analyses were considered exploratory and hypothesis-generating rather than statistically inferential.

### Calculation of gene signature scores

To investigate the variations within the interferon (IFN)/JAK-STAT signaling module, we employed the AddModuleScore function in Seurat v5 to determine the IFN/JAK-STAT signature score for various cell types. Genes used for signature scoring were selected based on a comprehensive manual review of the literature on interferon signaling and downstream JAK-STAT pathway activation.

### Spatial transcriptomics sequencing using Visium platform

We obtained spatially-resolved gene expression data from our patient and one naïve ASS-ILD control patient using the Visium Spatial Gene Expression assay (Visium, 10X Genomics). Four 10-micrometer sections from formalin-fixed paraffin-embedded (FFPE) tissue blocks were collected and processed for RNA extraction, ensuring a DV200 RNA quality metric of at least 30%. The sections were stained with H&E for morphological analysis and imaged. Probes were hybridized overnight, ligated, and treated with analyte transfer, RNA digestion, and probe release. DNA probes were captured on gene expression chips, followed by polymerase chain reaction (PCR) extension, elution, and transfer to EP tubes. Library construction was confirmed via quantitative PCR (qPCR) and Sample Index PCR (SI-PCR), then purified and assessed for quality. The cDNA library was sequenced on the Illumina NovaSeqX platform, providing data for downstream analysis.

### Spatial transcriptomic analysis using Visium HD platform

In order to obtain a more accurate tissue boundaries and alignment of the microscope image to spatial transcriptome data, manual alignment was conducted using Visium HD Manual Alignment Tool within Loupe Browser (v8.0.0). Then Space Ranger v3.0 was applied for the alignment of sequenced data to the annotated GRCh38 reference transcriptome Human Probe Set v2 from 10X Genomics. The 8 um binned data, recommended by 10X Genomics, were selected for downstream analysis. Seurat v5 (7) was used for bioinformatic analysis, with quality control excluding the top and bottom 2.5% of UMIs and genes per barcode as potential outliers, a similar workflow which was adopted in another Visium HD study (9). Considering the large size of the spatial transcriptome dataset, sketch-based analysis was adopted by subsampling 50,000 cells from each group. Routine data normalization and unsupervised clustering were conducted using Seurat. DEGs were identified via the MAST test with a min.pct of 0.1, adjusted *p-value* < 0.05, and average log_2_FoldChange > 0.5, and used for manual cluster annotation with ACT (http://xteam.xbio.top/ACT/) (10). Additional analyses, similar to those in the scRNA-seq study, were performed utilizing the same workflow as previously described.

## Results

### Characteristics of systemic inflammatory response

**Figure 2:**
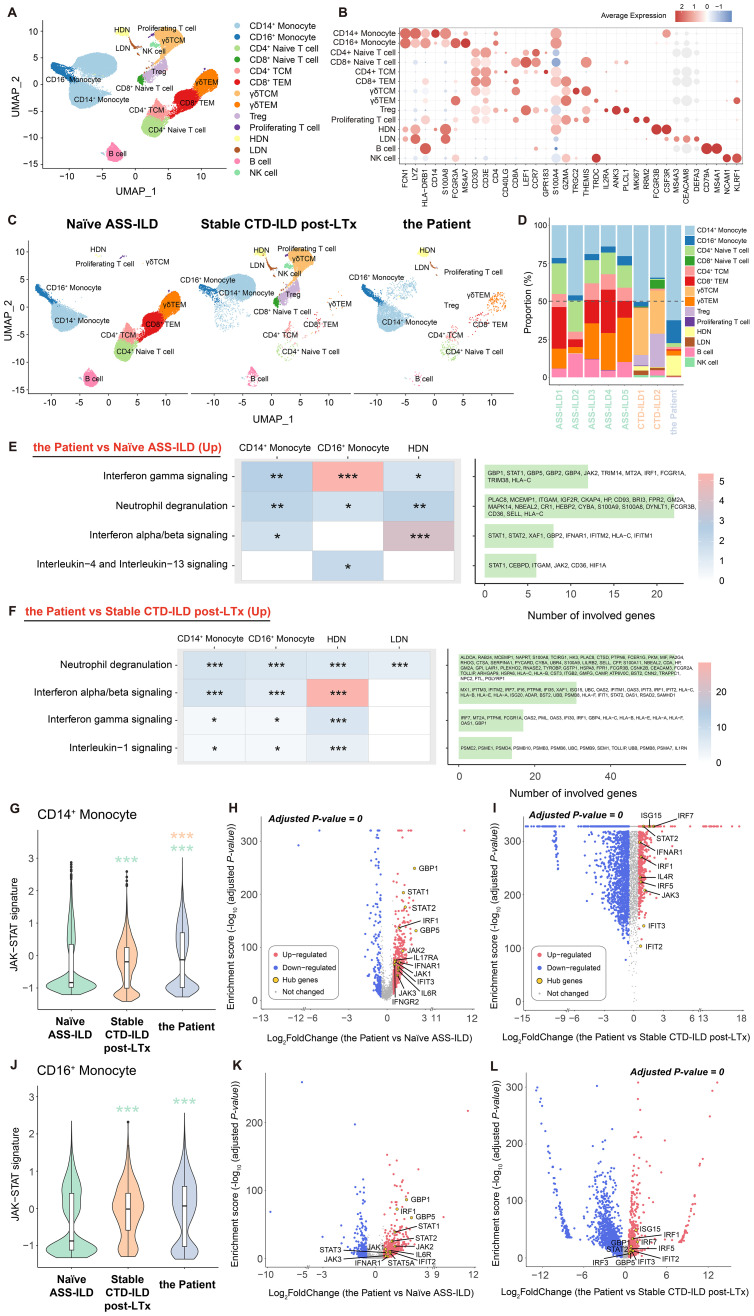
Comparative scRNA-seq analysis of the patient, five naïve ASS-ILD patients, and two clinically stable CTD-ILD patients post-LTx. **(A)** Unsupervised UMAP plot of PBMCs. TCM, central memory T cells; TEM, effector memory T cells; γδTCM, gamma-delta central memory T cells; γδTEM, gamma-delta effector memory T cells; Treg, regulatory T cells; HDN, high-density neutrophils; LDN, low-density neutrophils. **(B)** Expression levels of canonical marker genes across 14 distinct cell types. **(C)** UMAP plot of PBMCs under three conditions: naïve ASS-ILD patients (*left*), clinically stable post-LTx patients (*middle*) and the patient under investigation (*right*). **(D)** Distribution of 14 cells among different patients. **(E, F)** Reactome pathway enrichment analyses of **(E)** CD14^+^ monocytes, CD16^+^ monocytes, and HDNs from the patient versus naïve ASS-ILD patients, and **(F)** CD14^+^ monocytes, CD16^+^ monocytes, HDNs, and LDNs from the patient versus clinically stable CTD-ILD patients post-LTx. Genes contributing to each enriched pathway are indicated. Heatmap color intensity represents the enrichment score (defined as -log_10_ (*q-value*)), and asterisks denote different levels of enrichment: * represents *q-value* < 0.05, ** represents *q-value* < 0.01, and *** represents *q-value* < 0.001. **(G–L)** JAK-STAT gene signature scores (*left*) and volcano plot (*middle* and *right*) for CD14^+^ monocytes **(G–I)** and CD16^+^ monocytes **(J–L)**. JAK-STAT gene signature scores were compared at the single-cell level using pairwise two-sided Mann–Whitney U tests with Benjamini–Hochberg correction. Cell numbers were as follows: CD14^+^ monocytes (G)- naïve ASS-ILD (n = 6,044), stable CTD-ILD (n = 8,889), and the patient (n = 3,072); CD16^+^ monocytes (J)- naïve ASS-ILD (n = 1,136), stable CTD-ILD (n = 420), and the patient (n = 739). In the volcano plot, up-regulated DEGs, down-regulated DEGs and IFN/JAK-STAT related hub genes are highlighted in red, blue and yellow respectively.

To explore the inflammatory features of recurrent ASS in the post-LTx patient, we performed scRNA-seq on PBMCs using the 10X Genomics platform. After filtering out poor-quality cells and platelet markers, we integrated high-quality cells into a comparable dataset. This analysis revealed 14 major cell types (**Figures 2A, B**).

Compared to naïve ASS-ILD patients and clinically stable CTD-ILD patients post-LTx, this patient showed a higher proportion of CD14^+^ and CD16^+^ monocytes, with reduced proportions of T cells, B cells, and natural killer (NK) cells (**Figures 2C, D**; **Figure S5**). DEG analysis of upregulated genes was performed in CD14^+^ monocytes, CD16^+^ monocytes, and high-density neutrophils (HDNs) from this patient relative to naïve ASS-ILD patients. Exploratory pathway enrichment analysis suggested relative enrichment of interferon- and interleukin-related pathways, including interferon-gamma signaling, interferon-alpha/beta signaling, and interleukin-4/interleukin-13 signaling (**Figure 2E**). Consistently, a parallel DEG analysis of upregulated genes incorporating CD14^+^ monocytes, CD16^+^ monocytes, HDNs, and low-density neutrophils (LDNs), comparing this patient with clinically stable CTD-ILD patients post-LTx, reproduced similar enrichment patterns and suggested possible activation of interferon- and interleukin-related signaling pathways (**Figure 2F**).

To further quantify downstream signaling activity, a JAK-STAT signature score was calculated in CD14^+^ and CD16^+^ monocytes based on canonical pathway genes (including *JAK1, JAK2, TYK2, JAK3, STAT1, STAT2, STAT3, STAT4, STAT5A, STAT5B, STAT6*). Both monocyte subsets exhibited significantly elevated JAK-STAT signature scores (**Figures 2G, J**), supporting the observation of increased pathway-related transcriptional signatures. In line with these results, volcano plot analysis demonstrated marked up-regulation of key interferon/interleukin/JAK-STAT axis components, including JAK1, JAK2, STAT1, STAT2, STAT3, IL4R, IL6R, IRF1 and IFNGR2 in CD14^+^ and CD16^+^ monocytes from this patient compared with both naïve ASS-ILD patients (**Figures 2H, K**) and clinically stable CTD-ILD patients post-LTx (**Figures 2I, L**). Collectively, these exploratory analyses suggest relative enrichment of interferon-, interleukin-, and JAK-STAT-related signaling programs in this patient.

Meanwhile, the observed enrichment of neutrophil activation and degranulation pathways (**Figures 2E, F**) is consistent with, and may partly account for, the significantly elevated WBC levels in the patient’s peripheral blood.

### Change in treatment and follow-ups

After a multidisciplinary discussion involving specialists from the Department of Pulmonary and Critical Care Medicine, the Department of Lung Transplantation, and the Department of Rheumatology, we decided to increase the glucocorticoid dosage short-term (MP 120 mg/d for 3 days followed by rapid tapering). Considering the activation of the mononuclear phagocyte system in the patient, tacrolimus was replaced with cyclosporine A (target trough concentration 120–150 ng/ml), which is more widely used in macrophage activation syndrome (MAS) (11). Given the relative activation of the interferon and JAK-STAT pathways in the patient’s activated monocytes, we replaced MPA with the Janus kinase inhibitor (JAKi) tofacitinib at 5 mg twice daily.

After implementing the above treatment, the patient’s fever was controlled. One month later, the WBC count decreased to 14.54×10^9^/L, CK dropped to 654 U/L, CRP fell below detectable levels (2.5 mg/L), ferritin decreased to 843.1 ng/ml, and the PaO_2_/FiO_2_ ratio improved to 318 mmHg. Although the patient developed a urinary tract infection with *Klebsiella pneumoniae*, it was promptly managed with anti-infective treatment. A follow-up chest CT six months later showed significant resolution of the interstitial lesions in both lungs.

**Figure 3:**
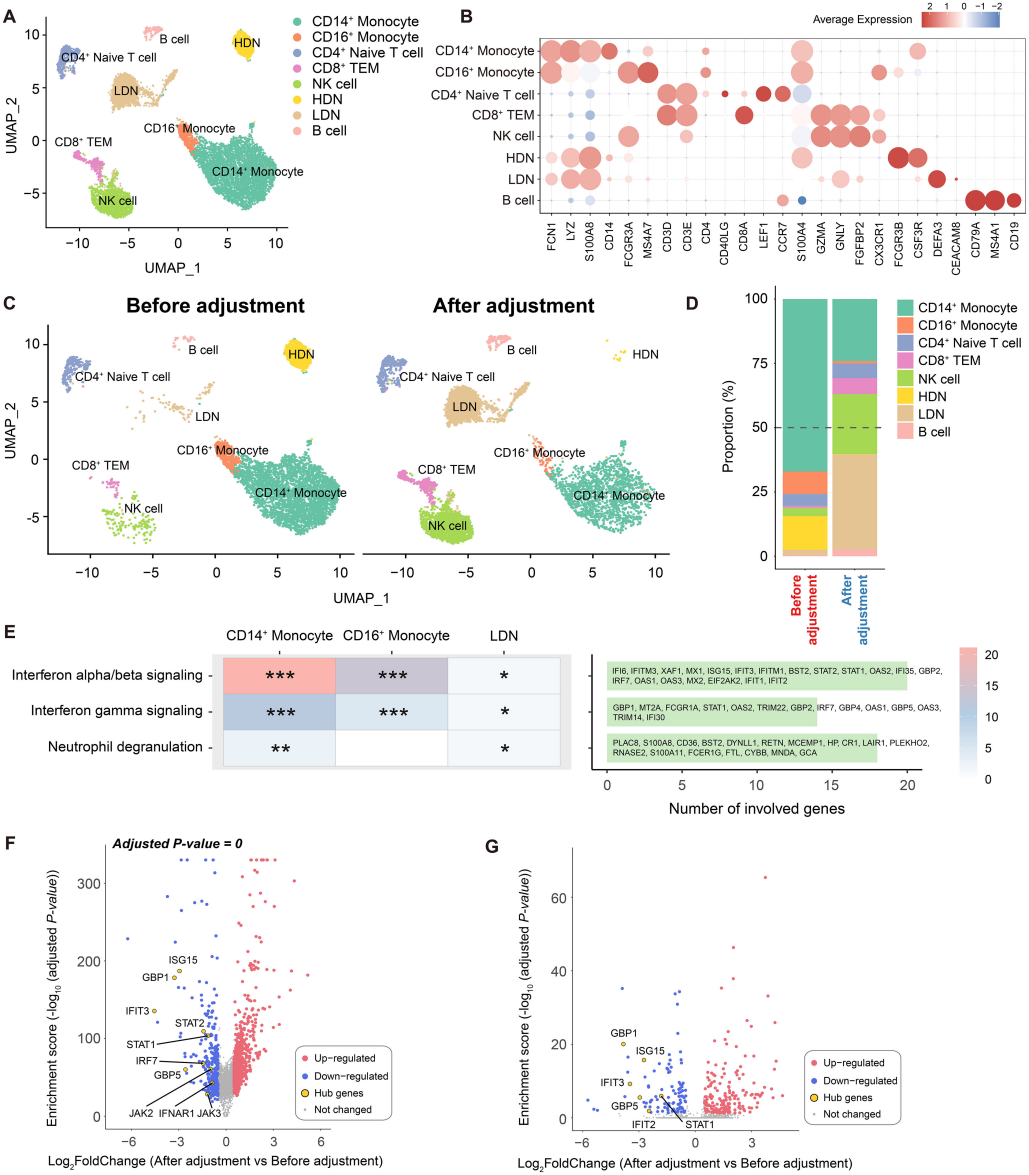
Comparative scRNA-seq profiling of the patient before and after adjustment. **(A)** Unsupervised UMAP plot of PBMCs. TEM, effector memory T cells; HDN, high-density neutrophils; LDN, low-density neutrophils. **(B)** Expression levels of canonical marker genes across 8 cell types. **(C)** UMAP plot of PBMCs under two conditions: after adjustment (*right*) and before adjustment (*left*). **(D)** Distribution of 8 cell types within the patient before (*left*) and after (*right*) adjustment. **(E)** Reactome pathway enrichment analyses for CD14^+^ monocytes, CD16^+^ monocytes, and LDNs. Cell colors within the heatmap represent the enrichment score (defined as -log_10_ (*q-value*)), and asterisks indicate different levels of enrichment: * represents *q-value* < 0.05, ** represents *q-value* < 0.01, and *** represents *q-value* < 0.001. **(F, G)** Volcano plot labeled with up-regulated DEGs (red), down-regulated DEGs (blue) and IFN/JAK-STAT related hub DEGs (*yellow*) in CD14^+^ monocytes **(F)** and CD16^+^ monocytes **(G)**.

At 84 weeks post-transplant, while the patient was in stable condition, we performed a follow-up scRNA-seq based on similar workflow to the one introduced before, which led to the identification of 8 cell types (**Figures 3A, B**) based on canonical markers. Compared to before the treatment adjustment, the proportion of CD14^+^ and CD16^+^ monocytes appear to have decreased, while T cells, NK cells, and B cells have increased (**Figures 3C, D**; **Figure S5**). Of note, following treatment adjustment, LDNs became more prominent in contrast to the predominance of HDNs observed prior to adjustment, characterized by high expression of *S100A8, S100A9* and *DEFA3* (**Figures 3B, D**; **Figure S5**).

In the functional enrichment analysis of DEGs, the interferon pathway, which was significantly up-regulated before the treatment adjustment, appeared to be significantly down-regulated in the subcluster of CD14^+^, CD16^+^ monocytes and LDNs (**Figure 3E**). Several related genes in the interferon pathway were also significantly down-regulated in CD14^+^ and CD16^+^ monocyte (**Figures 3F, G**). These findings suggested reduced interferon-related transcriptional signatures following the treatment adjustment.

Interestingly, at 86 weeks post-transplant, during a routine follow-up, the patient developed positive PRA, including newly emerged human leukocyte antigen (HLA) class II antibodies. High-resolution epitope analysis revealed moderately positive DQ2 (MFI 6193), a non-donor-specific antibody. Despite this, the patient was diagnosed with acute antibody-mediated rejection (AMR), potentially related to the treatment adjustment. At that time, the patient’s temperature, WBC count (11.59×10^9^/L), CK (279 U/L), and CRP (<2.5 mg/L) were stable. The patient was treated with intravenous immunoglobulin (IVIG) and resumed MPA (enteric-coated mycophenolate sodium (EC-MPS) 540 mg/day) to manage AMR. Given the use of four anti-inflammatory and immunosuppressive agents, the tofacitinib dosage was reduced to 5 mg once daily. At 95 weeks post-transplant, the patient experienced another urinary tract infection. However, laboratory tests showed stable CK (322 U/L) and ferritin (409.8 ng/ml) levels, and a follow-up chest CT showed continued improvement in interstitial lesions, with the PaO_2_/FiO_2_ ratio increasing to 373 mmHg.

**Figure 4:**
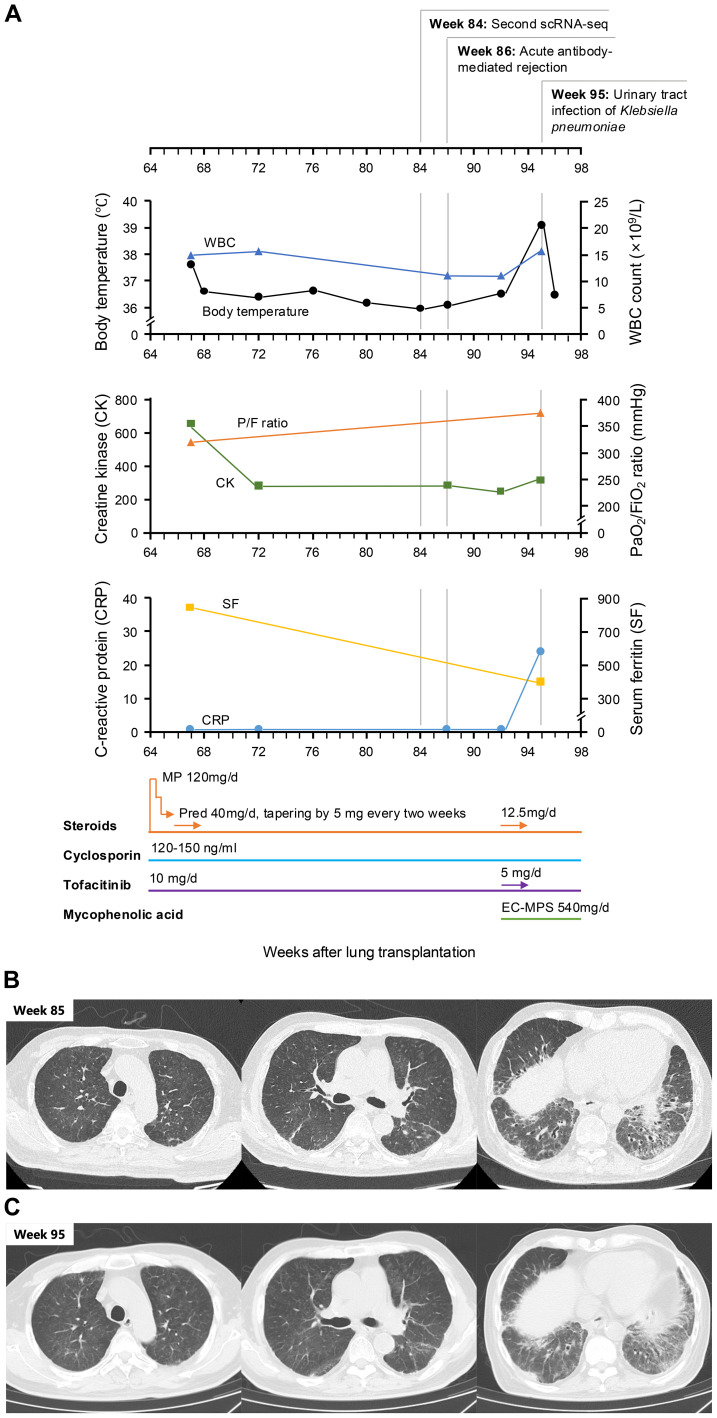
Clinical manifestations and immunosuppressive regimen after treatment adjustment based on scRNA-seq analysis. **(A)** Key clinical events, changes in laboratory parameters, and treatment regimens. Accompanying chest CT imaging changes at 85 **(B)**, and 95 **(C)** weeks post-transplantation. scRNA-seq, single-cell RNA sequencing; WBC, white blood cell; Pred, prednisone; MP, methylprednisolone; EC-MPS, enteric-coated mycophenolate sodium.

The clinical manifestations, key events, chest imaging changes, and immunosuppressive regimen after the treatment adjustment are presented in **Figure 4**.

### Characteristics of spatial transcriptomic analysis

**Figure 5:**
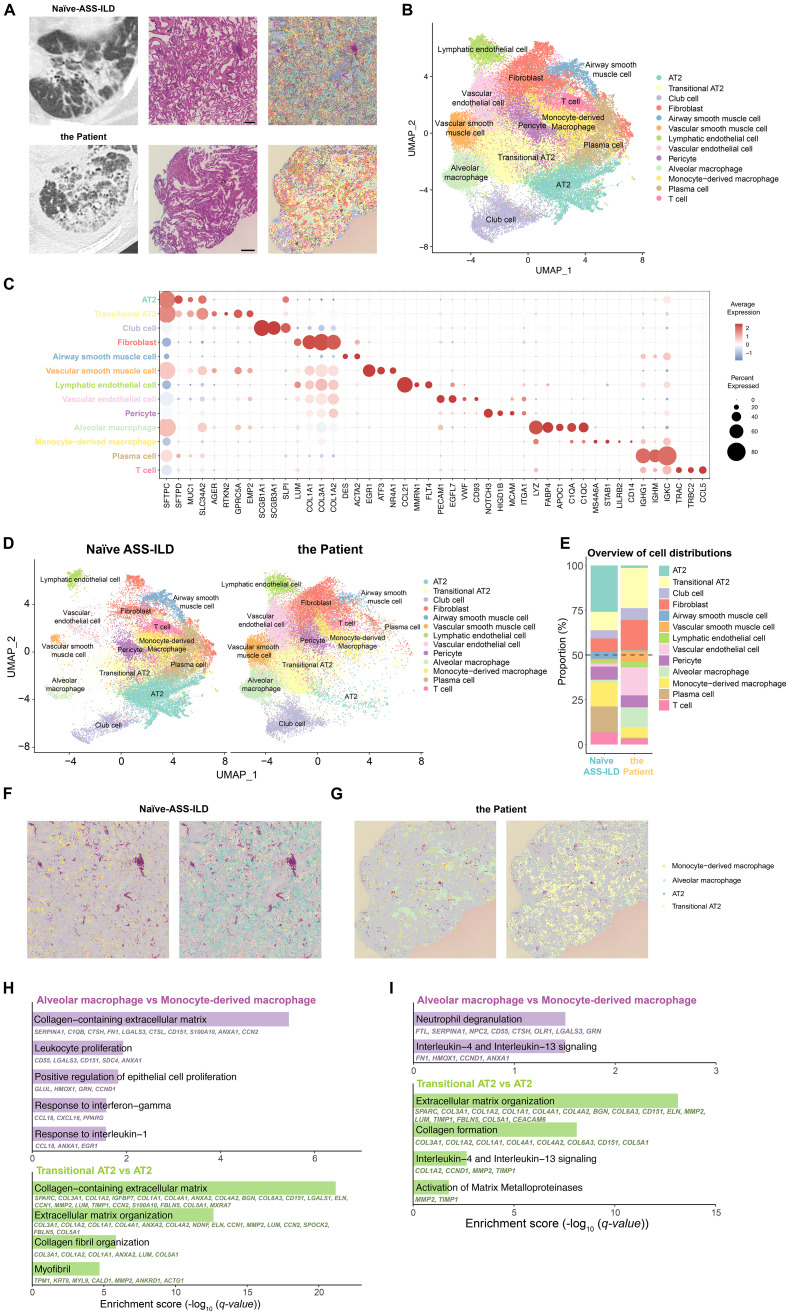
Spatial transcriptome analysis between the patient and one naïve ASS-ILD patient using 10X Visium HD. **(A)** Chest imaging, H&E staining of lung biopsy from the corresponding regions (scale bars, 200μm), and cellular annotation. The cell colors here correspond to the cell colors denoted in **Figure 5B**. **(B)** Unsupervised UMAP plot of subsampled lung tissue cells (sketch-based analysis). AT2, Type II alveolar cells. **(C)** Expression levels of markers for 13 cell types. **(D)** UMAP plot under two conditions: with LTx (*right*) and without LTx (*left*). **(E)** Distribution of 13 cells between the patient (*right*) and one naïve ASS-ILD patient (*left*). **(F, G)** Spatial distribution of monocyte-derived macrophages and alveolar macrophages, and AT2 cells and transitional AT2 cells in the naïve ASS-ILD patient **(F)** and the patient under investigation **(G)**. **(H, I)** GO **(H)** and Reactome **(I)** pathway enrichment analyses conducted on the up-regulated DEGs between alveolar macrophages and monocyte-derived macrophages (upper panel) as well as transitional AT2 cells and AT2 cells (lower panel).

To further investigate the spatial landscape of lung tissues in this patient, compared to the control group, we performed 10X Visium HD spatial transcriptomic analysis on corresponding FFPE tissues (**Figure 5A**). Using sketch-based analysis in Seurat v5, 53 clusters were identified with 25 PC and a resolution of 1.5. To improve cluster annotation accuracy, we removed clusters with fewer than 500 cells (~0.5% of total cells) and fewer than 10 marker genes. As a result, 13 cell types were manually annotated based on DEGs from each cluster (**Figures 5B, C**).

Compared to a naïve ASS-ILD patient, this patient’s lung tissue showed a higher proportion of macrophages, while the proportions of plasma cells and T cells were reduced (**Figures 5D, E**). This finding aligns with the scRNA-seq data from the patient’s PBMCs. Notably, within the macrophage population, the patient had a greater proportion of alveolar macrophages, whereas the control patient had more monocyte-derived macrophages. Given that interstitial pneumonia is often associated with alveolar epithelial cell injury, we observed that this patient exhibited a higher proportion of transitional type II alveolar cells (AT2), which expressed both AT2 canonical markers (*SFTPC, SFTPD*) and type I alveolar cells (AT1) canonical markers (*AGER, RTKN2, GPRC5A*) (**Figures 5C, E**).

To further investigate the distinct roles of dominant cell types in the two conditions, we extracted upregulated DEGs between alveolar macrophages and monocyte-derived macrophages, as well as between transitional AT2 cells and AT2 cells, followed by GO and Reactome enrichment analyses (**Figures 5F–I**). Several interferon-related pathways were enriched. Additionally, pathways associated with neutrophil activation were also observed. These findings were in line with the scRNA-seq results. Notably, both cell types from the patient showed enrichment in pro-fibrotic pathways like extracellular matrix organization and collagen formation. These observations suggest a potential association between systemic inflammatory transcriptional programs and pro-fibrotic features within the lung microenvironment.

## Discussion

We report a case of a patient with ASS-ILD who underwent bilateral LTx and experienced early recurrence of the underlying disease post-transplant, representing a typical *in vivo* model of ASS-ILD recurrence. Through exploratory scRNA-seq profiling, we descriptively characterized the patient’s systemic inflammatory profile and, based on these findings, selected an appropriate targeted therapeutic strategy that successfully controlled disease progression. Furthermore, we discussed the differences between the patient’s systemic inflammation and the transcriptional characteristics of naïve ASS-ILD and clinically stable CTD-ILD post-LTx patients.

The patient’s immune background was complex, with ASS as the underlying disease, post-LTx, and triple immunosuppressive therapy (glucocorticoids, tacrolimus, and MPA). ASS-ILD itself is a rare cause that requires LTx, and recurrence of ASS after LTx has never been reported before. The recurrence of ASS during classic anti-rejection treatment poses significant challenges in adjusting the immunosuppressive regimen. scRNA-seq, a high-throughput tool, provided an efficient way to explore the patient’s immune characteristics. The results suggested relative activation of the interferon and JAK–STAT pathways. During induction with short-term high-dose glucocorticoids, tacrolimus and MPA were replaced with cyclosporine A and tofacitinib, after which clinical improvement was observed. This case offers new insights into identifying suitable targeted treatments for complex systemic inflammation. Previous reports have also demonstrated the utility of scRNA-seq in guiding therapy for severe drug reaction with eosinophilia and systemic symptoms (DRESS) with successful outcomes (12). These cases make us look forward to the possibility of identifying more suitable potential therapeutic targets through omics analysis in the future, which can be seen as a new viewpoint of precision medicine.

IIMs are characterized by activation of the type I interferon pathway, with anti-melanoma differentiation-associated protein 5 (MDA5) dermatomyositis and ASS being the most notable subtypes (13–15). Both conditions commonly present with ILD, a major factor affecting patient survival. While most anti-MDA5 dermatomyositis and ASS cases respond to immunosuppressants such as cyclophosphamide, MPA, and CNIs, some refractory cases, especially those with rapidly progressive ILD, show poor responses (16). Retrospective and prospective studies have demonstrated the effectiveness of JAKi like tofacitinib and baricitinib in treating these refractory IIMs (17–20).

In this case, the immune characteristics revealed by scRNA-seq indicated that even though the patient was receiving triple immunosuppressive therapy, there was still relative activation of the mononuclear phagocyte system and enrichment of interferon, JAK-STAT pathways compared to ASS patients without LTx. This inflammatory transcriptional profile may explain why the patient experienced a recurrence of naïve ASS-ILD within one-year post-transplant. This finding was quite unexpected; however, fortunately, it enabled us to select a more targeted anti-inflammatory strategy. First, given the activation of this pathway, JAKi was considered the most direct therapeutic option. Among the available JAKi, given the simultaneous activation of multiple interferon pathways in this patient and based on prior clinical experience, we selected tofacitinib because of its relatively broad spectrum of targets (JAK1, JAK2 and JAK3) (21). Second, because the patient was in a post-transplant state, CNIs were required as the backbone immunosuppressive regimen. As tacrolimus failed to control disease progression, CsA was chosen as a replacement. Compared with tacrolimus, CsA might have relatively greater theoretical and literature-supported advantages in regulating monocyte–macrophage inflammatory mediators, oxidative stress/mitochondria-related mechanisms, and hyperinflammatory syndromes such as MAS (22–24). Besides, considering the mechanisms of action, expected onset times of these agents, and the patient’s critical condition, short-term high-dose glucocorticoids were also administered concomitantly as induction therapy to achieve rapid disease control. However, the patient had previously undergone glucocorticoid intensification at 39 weeks post-transplant, when ASS and associated ILD initially deteriorated. Although transient clinical improvement was observed, disease control could not be maintained during glucocorticoid tapering under continued tacrolimus and MPA therapy. While causality cannot be established, this temporal pattern raises the possibility that the subsequent introduction of tofacitinib and replacement of tacrolimus with cyclosporine A may have contributed to the sustained disease stabilization observed thereafter.

However, even after identifying a suitable treatment, medication adjustments were still necessary. The patient’s history of severe pulmonary and urinary tract infections indicated that adding JAKi to the post-transplant triple immunosuppressive regimen might be difficult to tolerate. As a result, MPA was discontinued upon initiation of tofacitinib. However, the patient subsequently developed AMR five months later. Although data regarding the incidence of AMR specifically in LTx recipients with CTD-ILD remain scarce, studies in the general post-LTx population have reported AMR rates ranging from approximately 4% (definite) to 29% (clinical) (25, 26). MPA is a cornerstone of maintenance immunosuppression and plays a critical role in suppressing alloimmune activation and preventing the development of DSAs. While a causal relationship cannot be established from a single case, several observations warrant consideration. In this case, AMR occurred after withdrawal of MPA and initiation of tofacitinib, and improved following reintroduction of MPA. These findings suggest that, despite the broad immunomodulatory effects of JAKi, whether they can provide graft-protective effects comparable to those of conventional anti-rejection agents remains uncertain. Consistent with this concern, a pilot study in *de novo* kidney transplant recipients found that JAKi-based regimens did not consistently demonstrate superior protection against acute rejection compared with conventional immunosuppressive therapies (27). Taken together, these observations emphasize the importance of close monitoring for alloimmune responses when reducing or discontinuing conventional anti-rejection regimens in LTx recipients. In addition, patients receiving JAKi require careful surveillance for potential adverse events, including infections, bone marrow suppression and cytopenias, thromboembolic complications, and malignancies (28). In this case, the dose of tofacitinib was reduced after reintroduction of MPA to minimize the risk of infection associated with combined immunosuppressive therapy.

Additionally, we obtained a lung biopsy specimen during the patient’s active phase of ASS and performed spatial transcriptomic analysis. Under the background of systemic inflammation characterized by relative activation of interferon and JAK–STAT signaling pathways, we further investigated the inflammatory features of the lung tissue and compared them with those of naïve ASS-ILD patients. The spatial transcriptomic analysis suggested persistent interferon-related transcriptional programs within the lung tissue. Additionally, functional enrichment of DEGs revealed organ-specific characteristics of effector cells, such as macrophages and fibroblasts, with strong pro-fibrotic properties (29, 30). This observation suggests that systemic inflammation and organ-specific responses may diverge in certain diseases. In our patient, spatial transcriptomic analysis of lung tissue demonstrated activation of interferon and JAK-STAT signaling pathways within a pro-inflammatory and pro-fibrotic microenvironment. Recent studies have identified transitional AT2 cells as a maladaptive epithelial state associated with impaired AT2-to-AT1 differentiation during alveolar repair, epithelial stress responses, and aberrant epithelial–mesenchymal crosstalk in fibrotic lung diseases (31, 32). The enrichment of transitional AT2 cells observed in this case may therefore represent an exploratory signal of ongoing epithelial injury and remodeling activity rather than direct evidence of a causal pathogenic mechanism. Notably, following treatment adjustment with intensified anti-inflammatory and immunomodulatory therapy, the patient experienced clinical and radiographic improvement, suggesting that at least part of this inflammatory-fibrotic program may remain reversible. These findings provide additional insight into the dynamic interplay between inflammation, epithelial dysfunction, and fibrosis in ILD.

This suggests that systemic inflammation and organ responses may differ in specific diseases. In this case, the patient’s lung tissue exhibited a pro-fibrotic background due to inflammation mediated by the interferon and JAK-STAT pathways, potentially associated with impaired AT2-to-AT1 cell differentiation and fibrotic remodeling. However, this process seemed partially reversible with targeted anti-inflammatory treatment, providing further insight into the relationship between inflammation and fibrosis in ILD.

Interferon is closely associated with ILD. As a drug, interferon has been clearly documented to cause adverse effects leading to lung injury (33). Coronavirus disease 2019 (COVID-19) has also been shown to exhibit significant dysregulation of the interferon pathway, with irreversible pulmonary fibrosis identified as the primary cause of death (34). This pathway is additionally linked with other connective tissue disease-associated ILD. In progressive pulmonary fibrosis related to systemic sclerosis, elevated levels of interferon-regulated genes, upregulation of transforming growth factor-beta (TGF-β) regulated genes, and macrophage activation have been observed (35). For this patient, active interferon and JAK-STAT pathway mediated inflammation led to pulmonary inflammation and fibrosis. Targeted anti-interferon therapy partially alleviated interstitial pneumonia but failed to fully reverse the fibrosis, likely due to the complexity of interferon-regulated pathways and the relative irreversibility of established fibrotic lesions. However, this suggests that combining anti-fibrotic therapy with targeted anti-inflammatory treatment may minimize irreversible fibrosis, warranting further investigation.

This case-based study has several limitations. First, while scRNA-seq provided personalized immune profiling, its high cost limits its broader application, making it more suitable for refractory and complex cases. Additionally, selecting appropriate control groups is challenging. In this case, we used naïve ASS-ILD and clinically stable CTD-ILD post-LTx patients as controls, considering that immune status in autoimmune disease recurrence post-transplant is more complex than in healthy individuals, which may obscure key immune mechanisms. Second, although scRNA-seq suggested that JAKi might represent a potential treatment, the observed clinical improvement occurred following a series of multidrug treatment adjustments, including glucocorticoid augmentation, substitution of tacrolimus with cyclosporine A, and the introduction of tofacitinib. Therefore, the independent contribution of JAKi cannot be isolated, and the relative contribution of each treatment component to the clinical improvement remains uncertain. Third, spatial transcriptomic and PBMC scRNA-seq analyses representing the active phase of ASS-ILD were performed at different time points (39^th^ and 56^th^ week post-LTx). Consequently, similarities and differences between lung and peripheral immune signatures should be interpreted as exploratory and complementary observations rather than direct temporal associations. Finally, recurrence of ASS-ILD after LTx is rare. Although the comparisons between our patient and the small control groups in this study provide several insights, the generalizability of the findings remains uncertain. Further validation in larger cohorts of patients with recurrent ASS-ILD, regardless of LTx status, is warranted.

## Conclusion

We presented a complex case of early recurrence of ASS after bilateral LTx. Using scRNA-seq, we explored the patient’s systemic inflammatory characteristics and adjusted targeted therapy, leading to a favorable outcome. Additionally, spatial transcriptomic analysis was employed to examine the differences between systemic inflammation and lung tissue response. This case highlights the potential of transcriptomic analysis in identifying inflammatory features and therapeutic targets in rheumatic diseases’ complex immune states. However, further research is needed to clarify its clinical utility.

## Data Availability

The datasets presented in this study can be found in online repositories. The names of the repository/repositories and accession number(s) can be found below: https://www.ncbi.nlm.nih.gov/, GSE286227 https://www.ncbi.nlm.nih.gov/, GSE286228 https://www.ncbi.nlm.nih.gov/, GSE324889.

## Glossary

AMR: Antibody-mediated rejection
ASS: Anti-synthetase syndrome
ASS-ILD: Anti-synthetase syndrome associated interstitial lung disease
AT1: Type I alveolar cells
AT2: Type II alveolar cells
BALF: Bronchioalveolar lavage fluid
BSA: Bovine serum albumin
cDNA: Complementary DNA
CK: Creatine kinase
CNI: Calcineurin inhibitor
COVID-19: Coronavirus disease 2019
CRP: C-reactive protein
CT: Computed tomography
CTD-ILD: Connective tissue disease associated interstitial lung disease
DEG: Differentially expressed gene
DRESS: Drug reaction with eosinophilia and systemic symptoms
EC-MPS: Enteric-coated mycophenolate sodium
EJ: Glycyl-tRNA synthetase
FFPE: Formalin-fixed paraffin-embedded
GEM: Gel bead-in-emulsion
HDN: High-density neutrophil
HLA: Human leukocyte antigen
IFN: Interferon
IIM: Idiopathic inflammatory myopathy
IVIG: Intravenous immunoglobulin
JAKi: Janus kinase inhibitor
Jo-1: Histidyl-tRNA synthetase
LDN: Low-density neutrophil
LTx: Lung transplantation
MAS: macrophage activation syndrome
MDA5: Melanoma differentiation-associated protein 5
MMF: Mycophenolate mofetil
MP: Methylprednisolone
MPA: Mycophenolic acid
MSA: Myositis-specific autoantibody
NK cell: Natural killer cell
NSIP: Nonspecific interstitial pneumonia
OJ: Isoleucyl-tRNA synthetase
PBMC: Peripheral blood mononuclear cell
PBS: Phosphate-buffered saline
PC: Principal component
PCR: Polymerase chain reaction
PE150: Paired-end 150bp
PL-7: Threonyl-tRNA synthetase
PL-12: Alanyl-tRNA synthetase
PRA: Positive panel-reactive antibody
qPCR: Quantitative PCR
RT: Reverse transcription
scRNA-seq: Single-cell RNA sequencing
SI-PCR: Sample Index PCR
TBLB: Transbronchial lung biopsy
TBLC: Transbronchial lung cryobiopsy
TGF-β: Transforming growth factor-beta
tRNA: Transfer RNA
UMAP: Uniform Manifold Approximation and Projection
WBC: White blood cell
