# Supplementary material for: Targeted therapy against recurrent anti-synthetase syndrome associated interstitial lung disease after bilateral lung transplantation guided by transcriptomic analyses: a case-based study
Source: Front Immunol. 2026 Jul 22;17:1857240. doi: 10.3389/fimmu.2026.1857240 (PMC13438322; doi:10.3389/fimmu.2026.1857240)
Supplement: Supplementary file 1 [file Supplementaryfile1.docx]

**Supplementary Materials**

**Title: Targeted therapy against recurrent anti-synthetase syndrome associated interstitial lung disease after bilateral lung transplantation guided by transcriptomic analyses****: a case-based study**

**Authors:** Shiyao Wang, MD^1,2,†^, Qingya Shi, MD, PhD^1,3,†^, Wenxiu Xu, MD^1,2^, Haibo Li, PhD^1^, Lili Zhu, MD^1^, Mengyin Chen, MD^1,2^, Shifeng Li, PhD^1,2^, Lijuan Guo, MD^1,2^, Li Zhao, MD^1,2^, Bin Xing, MD^1,2^, Zhibo Liu, MD^1,2^, Yi Zhang, MD^1,2^, Shiwei Qumu, MD^1,2^, Yinan Hu, MD^1^, Lingyan You, MD^1,4^, Quanguo Li, MD^1,5^, Jing Sun, MD^1,2^, Sizhao Li, MD^6^, Min Liu, MD^7^, Ling Zhao, MD^8^, Ye Cui, PhD^9^, Huaping Dai, MD^1,*^, Wenhui Chen, MD^1,2,*^

**Affiliations:**

1. National Center for Respiratory Medicine; State Key Laboratory of Respiratory Health and Multimorbidity; National Clinical Research Center for Respiratory Diseases; Institute of Respiratory Medicine, Chinese Academy of Medical Sciences; Department of Pulmonary and Critical Care Medicine, Center of Respiratory Medicine, China-Japan Friendship Hospital, Beijing, China. 100029
2. National Center for Respiratory Medicine; State Key Laboratory of Respiratory Health and Multimorbidity; National Clinical Research Center for Respiratory Diseases; Institute of Respiratory Medicine, Chinese Academy of Medical Sciences; Department of Lung Transplantation, Center of Respiratory Medicine, China-Japan Friendship Hospital, Beijing, China. 100029
3. School of Medicine, Tsinghua Medicine, Tsinghua University, Beijing, China. 100084
4. Center of Respiratory and Pulmonary Vascular Disease, Fuwai Hospital, National Center for Cardiovascular Disease, Chinese Academy of Medical Sciences and Peking Union Medical College, Beijing, China. 100037
5. Department of Pulmonary and Critical Care Medicine, Weifang Respiratory Disease Hospital, Weifang No.2 Renmin Hospital, Weifang, Shandong, China. 261041
6. Department of Rheumatology, China-Japan Friendship Hospital, Beijing, China. 100029
7. Department of Radiology, China-Japan Friendship Hospital, Beijing, China. 100029
8. Department of Pathology, China-Japan Friendship Hospital, Beijing, China. 100029
9. Department of Immunology, School of Basic Medical Sciences, Capital Medical University, Beijing, China. 100069

^†^ These authors contributed equally.

**^*^Corresponding author:**

Prof. Huaping Dai, M.D.

Affiliate: Department of Pulmonary and Critical Care Medicine, Center of Respiratory Medicine, China-Japan Friendship Hospital

Address: 2 Yinghuayuan East Street, Chaoyang District, Beijing 100029, China

Email: daihuaping@ccmu.edu.cn

Dr. Wenhui Chen, M.D.

Affiliate: Department of Lung Transplantation, Center of Respiratory Medicine, China-Japan Friendship Hospital

Address: 47 Wenxueguan Road, Chaoyang District, Beijing 100029, China

Email: wenhuichen1004@sina.com

**Table S1 Clinical Characteristics of the Five Naïve ASS-ILD Patients Used as Controls in the PBMC scRNA-seq Analysis**

|  | **Patient 1** | **Patient 2** | **Patient 3** | **Patient 4** | **Patient 5** |
| --- | --- | --- | --- | --- | --- |
| **Age** | 44 | 54 | 56 | 70 | 57 |
| **Gender** | F | F | F | F | F |
| **Duration of symptoms before Dx** | 4 months | 3 months | 1 year | 1 year | 6 months |
| **Clinical manifestations** | | | | | |
| **SOB and cough** | + | + | + | + | - |
| **Myositis** | - | + | - | - | - |
| **Rash** | Mechanic’s hands | Mechanic’s hands | Mechanic’s hands | Gottron papules | Mechanic’s hands |
| **Dry eyes or dry mouth** | - | + | - | + | + |
| **Lab tests** | | | | | |
| **ANA pattern** | cytoplasmic speckled pattern | cytoplasmic speckled pattern | nuclear speckled pattern | cytoplasmic speckled pattern | cytoplasmic speckled pattern |
| **ANA titer** | 1:40 | 1:80 | 1:40 | 1:40 | 1:80 |
| **MSA** | Anti-EJ | Anti-EJ | Anti-EJ | Anti-PL-7 | Anti-PL-7 |
| **WBC, ×10⁹/L** | 5.58 | 7.46 | 3.55 | 6.32 | 5.44 |
| **CK, U/L** | 38 | 221 | 43 | 41 | 70 |
| **CRP, mg/L** | 1.18 | 1.7 | 0.75 | 3.46 | 2 |
| **SF, ng/ml** | 16.8 | 77 | NA | NA | 150.1 |
| **LDH, U/L** | 151 | 328 | 163 | 169 | 179 |
| **PFR, mmHg** | 457 | 429 | 424 | 276 | 410 |
| **Pulmonary function tests** | | | | | |
| **FVC, (L, % predicted)** | 2.31 (73.0) | 1.55 (75.8) | 1.91 (69.9) | 1.63 (72.4) | 3.10 (108.7) |
| **FEV_1_/FVC, (%)** | 84.79 | 84.07 | 85.65 | 82.05 | 78.89 |
| **DLco, (% predicted)** | 58.3 | 55.1 | 64.5 | 69.7 | 89 |
| **Pathological pattern** | NSIP+OP | NSIP | NSIP+OP | NSIP | NSIP |

Abbreviations: ANA, antinuclear antibody; Anti-EJ, anti-glycyl tRNA synthetase antibody; Anti-PL-7, anti-threonyl tRNA synthetase antibody; ASS-ILD, anti-synthetase syndrome-associated interstitial lung disease; CK, creatine kinase; CRP, C-reactive protein; DLco, diffusing capacity of the lung for carbon monoxide; Dx, diagnosis; FEV₁, forced expiratory volume in one second; FVC, forced vital capacity; LDH, lactate dehydrogenase; MSA, myositis-specific antibody; NA, not available; NSIP, nonspecific interstitial pneumonia; OP, organizing pneumonia; PBMC, peripheral blood mononuclear cell; PFR, PaO₂/FiO₂ ratio; scRNA-seq, single-cell RNA sequencing; SF, serum ferritin; SOB, shortness of breath; WBC, white blood cell count.

Symbols: “+” indicates presence of the clinical manifestation; “−” indicates absence of the clinical manifestation.

**Table S2 Clinical Characteristics of the Two Stable CTD-ILD Patients Post-LTx Used as Controls in the PBMC scRNA-seq Analysis**

|  | **Patient 1** | **Patient 2** |
| --- | --- | --- |
| **Age** | 52 | 69 |
| **Gender** | F | F |
| **CTD-ILD diagnosis** | ASS-ILD | UCTD-ILD |
| **Indication for LTx** | PPF with endstage ILD  [FVC 0.63L (23.1%), DLco 10.5%] | AE-ILD, VV-ECMO dependent |
| **Time from LTx to PBMC sampling** | 3 months | 15 months |
| **Clinical status** | Stable | Stable |
| **Evidence of recurrence** | No | No |
| **Acute rejection** | No | No |
| **Active infection** | No | No |
| **Maintenance immunosuppression** | Prednisone + TAC + MPA | Prednisone + TAC + MPA |
| **Pulmonary function tests** | | |
| **FVC, (L, % predicted)** | 1.53(57.9) | 1.51(71.5) |
| **FEV_1_/FVC, (%)** | 98.6 | 83.4 |
| **DLco, (% predicted)** | 68.9 | 54.7 |

Abbreviations: AE-ILD, acute exacerbation of interstitial lung disease; ASS-ILD, anti-synthetase syndrome-associated interstitial lung disease; CTD-ILD, connective tissue disease-associated interstitial lung disease; DLCO, diffusing capacity of the lung for carbon monoxide; FEV₁, forced expiratory volume in one second; FVC, forced vital capacity; ILD, interstitial lung disease; LTx, lung transplantation; MPA, mycophenolate mofetil; PBMC, peripheral blood mononuclear cell; PPF, progressive pulmonary fibrosis; scRNA-seq, single-cell RNA sequencing; TAC, tacrolimus; UCTD-ILD, undifferentiated connective tissue disease-associated interstitial lung disease; VV-ECMO, venovenous extracorporeal membrane oxygenation.

**Table S3 Post-QC Metrics for scRNA-seq**

| ***Module1***: the patient before adjustment (week 56) vs. five naïve ASS-ILD and two stable post-LTx CTD-ILD controls | |
| --- | --- |
| **Samples** | **Number of high-quality cells per sample** |
| the Patient (week 56) | 4915 |
| ASS-ILD 01 | 5341 |
| ASS-ILD 02 | 3839 |
| ASS-ILD 03 | 6747 |
| ASS-ILD 04 | 5826 |
| ASS-ILD 05 | 5034 |
| CTD-ILD 01 | 11864 |
| CTD-ILD 02 | 8405 |
| ***Module2*: the patient before (week 56) vs. after (week 84) regimen adjustment** | |
| **Samples** | **Number of high-quality cells per sample** |
| the Patient (week 56) | 4922 |
| the Patient (week 84) | 6576 |

Abbreviations: ASS-ILD, anti-synthetase syndrome-associated interstitial lung disease; CTD-ILD, connective tissue disease-associated interstitial lung disease; LTx, lung transplantation

**Table S4 QC Metrics for Visium HD Spatial Transcriptomics**

| **Parameters** | **the Patient** | **Naïve ASS-ILD** |
| --- | --- | --- |
| Number of bins | 122,949 | 263,121 |
| Median genes per bin | 198 | 44 |
| Median total UMI per bin | 220 | 47 |
| Tissue coverage fraction (%) | 19.1 | 45.2 |

Abbreviations: ASS-ILD, anti-synthetase syndrome-associated interstitial lung disease; UMI, unique molecular identifier. Median per-bin metrics were calculated from QC-filtered Seurat objects; the tissue coverage fraction was obtained from the Space Ranger metrics_summary output (Fraction of Bins Under Tissue, 8 µm).

**Table S5 Canonical Markers for Manual Cell-type Annotation**

| **scRNA-seq cell types** (related to Figure 2B & Figure 3B) | |
| --- | --- |
| **Cell types** | **Canonical markers** |
| CD14^+^ Monocyte | FCN1, LYZ, S100A8, CD14 |
| CD16^+^ Monocyte | FCN1, LYZ, FCGR3A, MS4A7 |
| CD4+ Naive T cell | CD3D, CD3E, CD4, LEF1, CCR7 |
| CD8+ Naive T cell | CD3D, CD3E, CD8A, LEF1, CCR7 |
| CD4+ TCM | CD3D, CD3E, CD4, GPR183, S100A4 |
| CD8+ TEM | CD3D, CD3E, CD8A, S100A4, GZMA |
| γδTCM | CD3D, CD3E, TRGC2, THEMIS |
| γδTEM | CD3D, CD3E, TRDC |
| Treg | CD3D, CD3E, CD4, IL2RA, ANK3, PLCL1 |
| Proliferating T cell | CD3D, CD3E, MKI67, RRM2 |
| HDN | S100A8, FCGR3B, CSF3R |
| LDN | S100A8, DEFA1/DEFA3, CEACAM8 |
| B cell | CD79A, MS4A1 |
| NK cell | NCAM1, KLRF1 |
| **Spatial transcriptomics cell types (related to Figure 5C)** | |
| **Cell types** | **Canonical markers** |
| AT2 | SFTPC, SFTPD, MUC1, SLC34A2 |
| Transitional AT2 | SFTPC, SFTPD, MUC1, SLC34A2 |
| Club cell | SCGB1A1, SCGB3A1, SLPI |
| Fibroblast | LUM, COL1A1, COL3A1, COL1A2 |
| Airway smooth muscle cell | DES, ACTA2 |
| Vascular smooth muscle cell | EGR1, ATF3, NR4A1 |
| Lymphatic endothelial cell | CCL21, MMRN1, FLT4 |
| Vascular endothelial cell | PECAM1, EGFL7, VWF, CD93 |
| Pericyte | NOTCH3, HIGD1B, MCAM |
| Alveolar macrophage | LYZ, FABP4, APOC1, C1QA, C1QC |
| Monocyte-derived macrophage | LYZ, MS4A6A, STAB1, LILRB2, CD14 |
| Plasma cell | IGHG1, IGHM, IGKC |
| T cell | TRAC, TRBC2, CCL5 |

Abbreviations: AT2, alveolar type II epithelial cell; HDN, high-density neutrophil; LDN, low-density neutrophil; NK, natural killer; scRNA-seq, single-cell RNA sequencing; TCM, central memory T cell; TEM, effector memory T cell; Treg, regulatory T cell; γδTCM, γδ central memory T cell; γδTEM, γδ effector memory T cell.

**Table S6 Software Environment and Package Versions Used for Analysis and Visualization**

| **Software/Package** | **Purpose** | **Version** |
| --- | --- | --- |
| R | Base environment | 4.4.2 |
| Seurat | scRNA-seq & spatial analysis, clustering, DEG, AddModuleScore | 5.4.0 |
| harmony | Batch-effect correction | 1.2.4 |
| DoubletFinder | Doublet removal | 2.0.4 |
| MAST | Differential expression test | 1.32.0 |
| ReactomePA | Reactome pathway enrichment | 1.50.0 |
| clusterProfiler | GO pathway enrichment | 4.14.6 |
| ggplot2 | Visualization | 4.0.1 |
| ggpubr | Violin-plot statistics | 0.6.2 |
| rstatix | Statistical analysis | 0.7.3 |
| Space Ranger | Spatial read alignment | 3.0 |
| Loupe Browser | Visium HD manual alignment | 8.0.0 |
| ACT (web server) | Spatial cluster annotation | n/a (web server) |

Abbreviations: DEG, differentially expressed gene; GO, Gene Ontology; scRNA-seq, single-cell RNA sequencing.

**Supplementary Figures**

**Figure S1. Pulmonary function and radiological findings of the patient before LTx.** Serial pulmonary function tests demonstrated progressive respiratory deterioration before LTx, fulfilling the criteria for progressive fibrosing interstitial lung disease (PF-ILD). HRCT images obtained 1 month before LTx demonstrated bilateral eticulation, traction bronchiectasis, and basal-predominant fibrosis, consistent with fibrotic nonspecific interstitial pneumonia (fNSIP). Abbreviations: DLCO, diffusing capacity of the lung for carbon monoxide; FEV₁, forced expiratory volume in one second; FVC, forced vital capacity; fNSIP, fibrotic nonspecific interstitial pneumonia; HRCT, high-resolution computed tomography; LTx, lung transplantation; y, year(s); m, month(s).

**Figure S2.** Timeline of sample collection and major clinical events of the Patient.

Abbreviations: MP, methylprednisolone; Pred, prednisone; TAC, tacrolimus; CsA, cyclosporine A; MPA, mycophenolic acid; TOFA, tofacitinib; PBMC, peripheral blood mononuclear cell; scRNA-seq, single-cell RNA sequencing; TBLB, transbronchial lung biopsy; AMR, antibody-mediated rejection; IVIG, intravenous immunoglobulin.

**Figure S3. HRCT images of the five naïve ASS-ILD patients included as controls in the PBMC scRNA-seq analysis.**

Abbreviations: ASS-ILD, anti-synthetase syndrome-associated interstitial lung disease; HRCT, high-resolution computed tomography; PBMC, peripheral blood mononuclear cell; scRNA-seq, single-cell RNA sequencing.

**Figure S4. HRCT images of the two stable CTD-ILD patients post-LTx used as controls in the PBMC scRNA-seq analysis.**

Abbreviations: CTD-ILD, connective tissue disease-associated interstitial lung disease; HRCT, high-resolution computed tomography; LTx: lung transplantation; PBMC, peripheral blood mononuclear cell; scRNA-seq, single-cell RNA sequencing.

**Figure S5.** **Comparison of the proportions of different cell types across groups based on scRNA-seq.** (**A**) Proportion of 14 types of cells annotated from scRNA-seq analysis between five naïve ASS-ILD patients without LTx, two clinically stable CTD-ILD patients with LTx and our patient (corresponding to **Figure 2**). (**B**) Proportion of 8 types of cells annotated from scRNA-seq analysis of our patient before and after adjustment (corresponding to **Figure 3**). Abbreviations: ASS-ILD, anti-synthetase syndrome-associated interstitial lung disease; CTD-ILD, connective tissue disease-associated interstitial lung disease; HDN, high-density neutrophil; LDN, low-density neutrophil; LTx, lung transplantation; NK, natural killer cell; scRNA-seq, single-cell RNA sequencing; TCM, central memory T cell; TEM, effector memory T cell; Treg, regulatory T cell; γδTCM, γδ central memory T cell; γδTEM, γδ effector memory T cell.
